# Chemotranscriptomic Profiling Defines Drug-Specific Signatures of the Glycopeptide Antibiotics Dalbavancin, Vancomycin and Chlorobiphenyl-Vancomycin in a VanB-Type-Resistant Streptomycete

**DOI:** 10.3389/fmicb.2021.641756

**Published:** 2021-02-25

**Authors:** Andy Hesketh, Giselda Bucca, Colin P. Smith, Hee-Jeon Hong

**Affiliations:** ^1^School of Pharmacy and Biomolecular Sciences, University of Brighton, Brighton, United Kingdom; ^2^Department of Biological and Medical Sciences, Oxford Brookes University, Oxford, United Kingdom

**Keywords:** glycopeptide, antibiotic, resistance, vancomycin, dalbavancin, chemotranscriptomics, *Streptomyces*

## Abstract

Dalbavancin, vancomycin and chlorobiphenyl-vancomycin share a high degree of structural similarity and the same primary mode of drug action. All inhibit bacterial cell wall biosynthesis through complexation with intermediates in peptidoglycan biosynthesis mediated via interaction with peptidyl-d-alanyl–d-alanine (d-Ala–d-Ala) residues present at the termini of the intermediates. VanB-type glycopeptide resistance in bacteria encodes an inducible reprogramming of bacterial cell wall biosynthesis that generates precursors terminating with d-alanyl–d-lactate (d-Ala–d-Lac). This system in *Streptomyces coelicolor* confers protection against the natural product vancomycin but not dalbavancin or chlorobiphenyl-vancomycin, which are semi-synthetic derivatives and fail to sufficiently activate the inducible VanB-type sensory response. We used transcriptome profiling by RNAseq to identify the gene expression signatures elucidated in *S. coelicolor* in response to the three different glycopeptide compounds. An integrated comparison of the results defines both the contribution of the VanB resistance system to the control of changes in gene transcription and the impact at the transcriptional level of the structural diversity present in the glycopeptide antibiotics used. Dalbavancin induces markedly more extensive changes in the expression of genes required for transport processes, RNA methylation, haem biosynthesis and the biosynthesis of the amino acids arginine and glutamine. Chlorobiphenyl-vancomycin exhibits specific effects on tryptophan and calcium-dependent antibiotic biosynthesis and has a stronger repressive effect on translation. Vancomycin predictably has a uniquely strong effect on the genes controlled by the VanB resistance system and also impacts metal ion homeostasis and leucine biosynthesis. Leaderless gene transcription is disfavoured in the core transcriptional up- and down-regulation taking place in response to all the glycopeptide antibiotics, while HrdB-dependent transcripts are favoured in the down-regulated group. This study illustrates the biological impact of peripheral changes to glycopeptide antibiotic structure and could inform the design of future semi-synthetic glycopeptide derivatives.

## Introduction

Glycopeptide antibiotics are important antibacterial agents for the treatment and control of infections caused by Gram-positive pathogens, particularly those that show resistance to other antibiotics. Vancomycin, a natural metabolite first discovered and isolated from cultures of the actinomycete *Amycolatopsis orientalis* (McCormick et al., 1955), is the pioneer compound in the glycopeptide antibiotic class. Approved for clinical use in 1958, it has since been joined by four additional structurally related natural and semi-synthetic glycopeptides, namely teicoplanin, oritavancin, dalbavancin and telavancin (reviewed in Butler et al., 2014; Blaskovich et al., 2018; Marcone et al., 2018). All these compounds currently find application in the treatment of staphylococcal, enterococcal and clostridial infections, but, as with all antibiotic chemotherapies, they are vulnerable to the development of resistance (Pootoolal et al., 2002).

High-level resistance to vancomycin occurs through an inducible mechanism that reprograms the biosynthesis of intermediates in bacterial cell wall peptidoglycan production (Bugg et al., 1991a, b; Walsh et al., 1996). The primary mode of action of glycopeptide antibiotics is the inhibition of peptidoglycan biosynthesis through complexation with biosynthetic intermediates possessing peptidyl-d-alanyl–d-alanine (d-Ala–d-Ala) residues (Barna and Williams, 1984). Complex formation prevents efficient incorporation of the intermediates into the mature peptidoglycan cell wall structure. Inducible resistance systems generate precursors terminating with d-alanyl–d-lactate (d-Ala–d-Lac) in preference to d-Ala–d-Ala and thereby enable continued peptidoglycan biosynthesis in the presence of vancomycin. Increased tolerance to vancomycin can also be achieved by non-inducible mechanisms. In *Staphylococcus aureus*, for example, increased cell wall thickness and changes to cell wall composition and turnover are associated with intermediate level glycopeptide antibiotic resistance (Hanaki et al., 1998a, b; Cui et al., 2003; Howden et al., 2010). Conversely, environmental factors can increase sensitivity to glycopeptide activity in highly resistant strains. The presence of high concentrations of inorganic phosphate (>0.25% w/v) sensitises *Streptomyces* species to vancomycin by hampering transcriptional induction of the resistance genes (Santos-Beneit and Martin, 2013; Santos-Beneit, 2018).

The emergence and spread of vancomycin resistant pathogens in hospitals around the world is an important healthcare concern. The 2019 Antibiotic Resistance Threats Report from the United States’ Centers for Disease Control and Prevention lists VanB-type vancomycin-resistant enterococci in their “serious threat” category (CDC, 2019). VanB-type resistance is defined by inducible resistance to vancomycin but susceptibility to teicoplanin and related analogues (unless vancomycin is also present). Routes to the production of new glycopeptide antibiotics with improved efficacy are continually being sought and include total chemical synthesis (Okano et al., 2014, 2017), chemical or chemoenzymatic derivatisation of glycopeptide scaffolds (Yarlagadda et al., 2015; Blaskovich et al., 2018; MishraI, Stolarzewicz et al., 2018; Tailhades et al., 2020), and the development of synthetic biology platforms (Xu et al., 2020). Successful modification of a glycopeptide structure might result from the generation of molecules with reduced ability to activate the known inducible glycopeptide resistance systems or the conferment of antibacterial activities independent of the interaction with d-Ala–d-Ala residues. There is, however, only a limited understanding of the impact that peripheral changes to the structure of glycopeptide antibiotics might have on their antibacterial activity.

**FIGURE 1:**
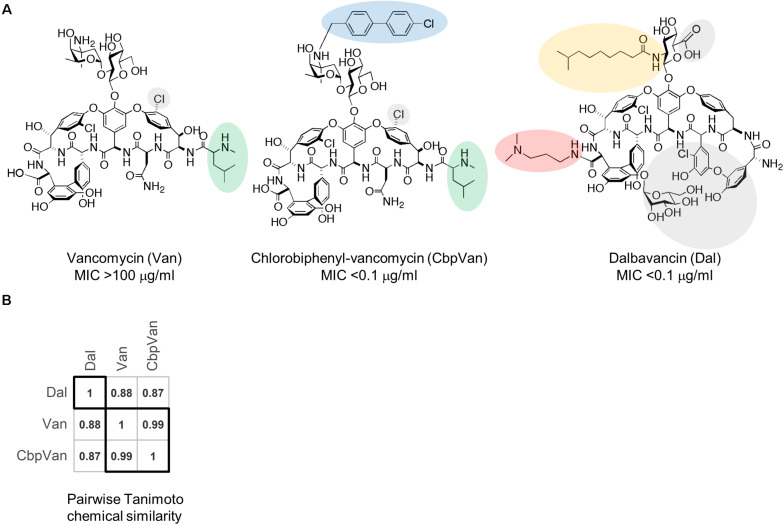
The glycopeptide antibiotics used in this study. Structures of vancomycin (Van), chlorobiphenyl-vancomycin (CbpVan) and dalbavancin (Dal), and their corresponding minimum inhibitory concentration (MIC) values against *S. coelicolor* in liquid cultures (Kwun and Hong, 2014) **(A)** and pairwise Tanimoto chemical similarity scores **(B)**. In **(A)**, distinctive structural features are highlighted, including the following that are also discussed in the text: N-methylleucine group (green); chlorobiphenyl group (blue); lipid side chain (orange); dimethylaminopropyl amide side chain (red).

Transcriptome profiling has previously been used as a method to define bacterial responses to large panels of chemically very diverse antibiotics in studies aimed at improving antibacterial discovery pipelines (Boshoff et al., 2004; Hutter et al., 2004; Jones et al., 2017; O’Rourke et al., 2020). Transcriptome profiles of the immediate response to antibiotic challenge, typically within 30-60 min, allowed classification of compounds into mechanism of action (MOA) groups which could be exploited to predict the MOA of new antibiotic candidates. Here we use a similar chemotranscriptomics approach to define the structure-activity relationships among a panel of three very closely related glycopeptide antibiotic structures – dalbavancin, vancomycin and chlorobiphenyl-vancomycin (Figure 1A) – using *Streptomyces coelicolor* as a biological activity reporter strain. *S. coelicolor* possesses a well-characterised VanB-type glycopeptide antibiotic resistance system that confers full protection against the natural product vancomycin but not against dalbavancin or chlorobiphenyl-vancomycin (Hong et al., 2004, 2005; Hutchings et al., 2006; Kwun and Hong, 2014). Vancomycin and chlorobiphenyl-vancomycin are visually very similar molecules and score highly on a pairwise Tanimoto chemical similarity index (Figure 1B). They differ, however, in the presence of the hydrophobic chlorobiphenyl group conjugated to the disaccharide moiety through the vancosamine sugar, as also found in oritavancin, which markedly increases its activity against *S. coelicolor* (Kwun and Hong, 2014). Dalbavancin is a lipoglycopeptide antibiotic and is the least similar of the three molecules (Figure 1). It is a semi-synthetic derivative of a teicoplanin-like natural product and is characterised by an extended lipophilic side chain attached to the monosaccharide moiety and by the presence of a dimethylaminopropyl amide side chain. The chemotranscriptomic profiling results define the impact of the structural diversity present in the glycopeptide antibiotics tested at the level of the bacterium’s transcriptional response. Functional interpretation of the different responses identifies the biological consequences of the differences in structure. A conserved response is also identified and discussed.

## Materials and Methods

### Bacterial Cultures

Spores of *S. coelicolor* M600 were germinated and grown to mid-log phase (OD450 nm approx. 0.5) in liquid NMMP medium as described previously (Hong et al., 2004, 2005; Tran et al., 2019). Biological duplicate cultures were treated at this point by adding vancomycin (Sigma), dalbavancin or chlorobiphenyl-vancomycin to a final concentration of 10 μg/ml. A vehicle-only control (dimethylsulphoxide) in which cultures received no antibiotic treatment was also applied. Cultures were incubated for a further 30 min before harvesting for total RNA extraction. The decision to use 10 μg/ml for all three antibiotics (which possess different MICs above and below this threshold) was based on Kwun and Hong, 2014 (Kwun and Hong, 2014) where this dose produced a strong cell wall stress transcriptional response in *S. coelicolor* (as measured by qRT-PCR of selected reporter genes) in the 30-60 min following treatment with a panel of 26 glycopeptide antibiotics. This was irrespective of whether the compounds used possessed MICs well below or well above 10 μg/ml. A treatment of 10 μg/ml over 30 min is therefore appropriate to capture the immediate response of interest.

### RNA Extraction and Sequencing

Total RNA was isolated from mycelium harvested from 5 ml liquid culture aliquots using the procedure previously described (Tran et al., 2019). RNA concentration, purity and integrity were determined using a NanoDrop 1C (NanoDrop Technologies) spectrophotometer and an Agilent 4200 TapeStation (Agilent Technologies). All samples passed purity quality control checks and exhibited mid-range RNA Integrity Number values. For each sample, 2 μg aliquots were subjected to ribosomal RNA depletion using the Illumina Ribo-Zero rRNA Removal Kit (Bacteria). Successful depletion was verified by analysis on the Agilent TapeStation. Strand-specific sequencing libraries were prepared using the TruSeq stranded mRNA library prep and TruSeq RNA single indexes set A (Illumina). All libraries were checked on the Agilent TapeStation using a D1000 High Sensitivity tape. Libraries were quantified, pooled and subjected to single-end 150 bp sequencing using an Illumina NextSeq500 sequencer with the Med OutPut Illumina kit 1 × 150 cycles. The raw sequencing run was demultiplexed on the Illumina BaseSpace platform, generating fastq.gz files for subsequent analysis. This data is available in the ArrayExpress database^1^ under accession number E-MTAB-9846.

### Data Analysis

Raw fastq.gz sequencing data was quality checked using fastqc (v.0.11.8) (Andrews, 2010) and multiqc (v1.7) (Ewels et al., 2016). Gene transcript abundances were determined by pseudoalignment of the raw sequencing reads to a reference transcriptome using kallisto (Bray et al., 2016). The reference transcriptome was generated from the sequence and annotation in genome version GCF_000203835.1_ASM20383v1, plus all the short non-coding RNA sequences (sRNA) reported in Jeong et al. (2016). Raw count data were filtered to remove lowly expressed transcripts (682 removed), and the data were normalised and tested for differential expression using the limma voom pipeline (Ritchie et al., 2015). The limma TREAT approach was applied to test changes for significance against a 50% fold-change threshold, and all *p*-values were corrected for multiple testing using the Benjamini and Hochberg method (McCarthy and Smyth, 2009). Each glycopeptide antibiotic treatment was referenced to the common control condition. Functional enrichment tests were performed using clusterProfiler (Yu et al., 2012) and the gene ontology Biological Process annotation for *S. coelicolor* present in AnnotationHub (Morgan and Shepherd, 2020). For the enrichment analyses, genes were assigned to groups according to their combined response to each antibiotic (Supplementary Table 1). Analyses for under- or over-representation of leaderless transcripts and HrdB-dependent transcripts were performed using the fisher.test function in the R stats package (R Core Team, 2020). For this, the transcript groups that showed opposing regulation in response to any two antibiotics were first removed from the data (43 transcripts removed). Gene transcripts were assigned as originating from leaderless, or 5′-UTR leadered, transcription using data for primary transcription start sites published in Jeong et al. (2016). Operon structure was included in the assignments such that, for example, where a leaderless transcript covers transcription of a three gene operon, all three genes are classed as leaderless. Where necessary, operon structure was taken from Charaniya et al. (2007). Dependence of transcription on sigma HrdB was assigned using the data published in Smidova et al. (2019). Pairwise Tanimoto similarity scores for the glycopeptide antibiotic structures were obtained from ChEBI (Hastings et al., 2016).

## Results

**FIGURE 2:**
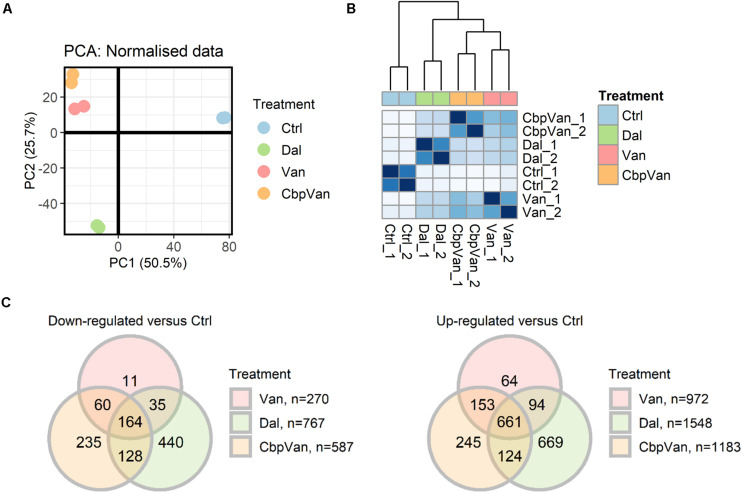
The transcriptional responses of *S. coelicolor* cells to exposure to vancomycin (Van), chlorobiphenyl-vancomycin (CbpVan) and dalbavancin (Dal). Principal components analysis (PCA) **(A)** and hierarchical clustering of Euclidean sample distances **(B)** indicate closer similarity between the data for Van and CbpVan compared to Dal. Differential expression analysis identifies gene transcripts significantly changing in abundance in response to one or more glycopeptide antibiotic compared to the control (Ctrl) **(C)**. In **(B)**, darker blue grid colours indicate closer similarity between pairs of samples.

The global transcriptional responses of *S. coelicolor* cells to vancomycin, chlorobiphenyl-vancomycin and dalbavancin were captured by treating liquid cultures grown to mid-log phase in a minimal medium for 30 min with 10 μg/ml of each antibiotic. This concentration is below the minimum inhibitory concentration (MIC) for vancomycin, but above that for chlorobiphenyl-vancomycin and dalbavancin (Figure 1A). Previous work defined 30-60 min as the time required to produce a maximal cell wall stress transcriptional response in *S. coelicolor* to 10 μg/ml of each of a panel of 26 glycopeptide antibiotics possessing MICs in the same 0.1 to 100 μg/ml range (Kwun and Hong, 2014). Antibiotic challenge studies in *Escherichia coli* and *S. aureus* have also considered a 30 min exposure time to be ideal for observing compound-specific responses while avoiding indirect effects (Jones et al., 2017; O’Rourke et al., 2020). The experiments were performed in biological duplicate, and the results after RNA sequencing were compared to those obtained from a similar vehicle-only control treatment. Unsupervised clustering of the normalised gene transcript abundance data indicates that exposure to all three glycopeptides generates transcriptional profiles distinct from the untreated cells, but that vancomycin and chlorobiphenyl-vancomycin produce responses more similar to each other than to the response to dalbavancin (Figures 2A,B). Differential expression analysis identified 1242, 1770 and 2315 transcripts significantly differently expressed (adj.P.Val ≤ 0.05) between antibiotic and control treatments for vancomycin, chlorobiphenyl-vancomycin and dalbavancin, respectively, comprising 14–28% of the transcriptome (Supplementary Data 1). Of these, significant up or down regulation in response to all three glycopeptides was conserved for 825 transcripts (∼10% of the transcriptome) (Figures 2C,D). A total of 3040 unique gene transcripts responded significantly to at least one of the antibiotics used.

### Differences in Glycopeptide Structure Modulate Activation of the VanB-Type Resistance System and Zinc Metabolism but Not Cell Wall Stress

**FIGURE 3:**
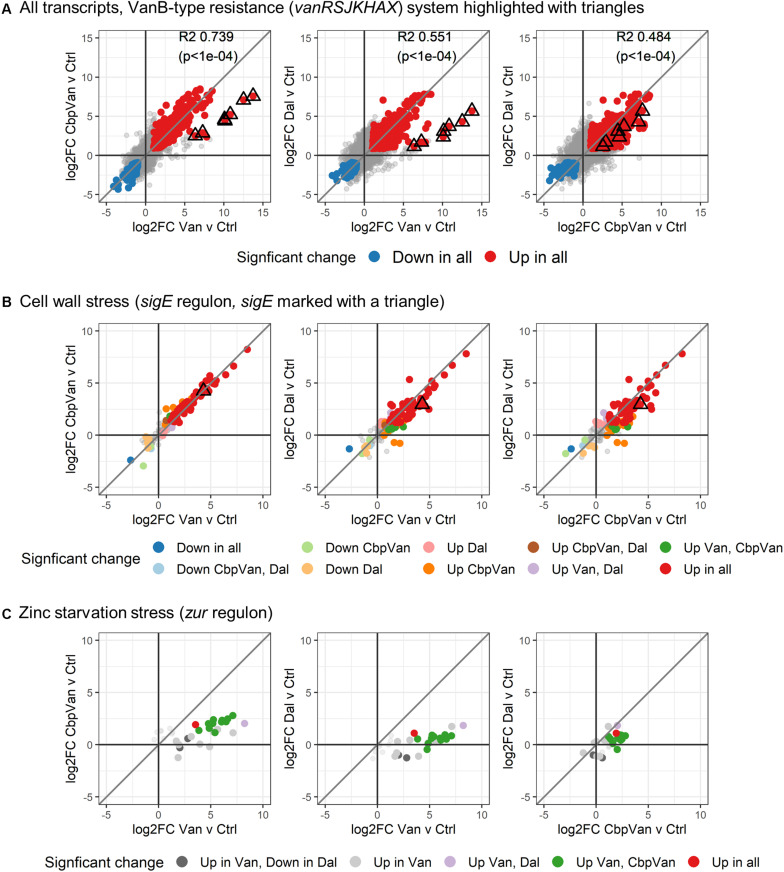
Differences in activation of the VanB-type resistance system **(A)**, the SigE-controlled cell wall stress response **(B)** and zinc starvation stress response **(C)**. In each panel, the diagonal line illustrates theoretical perfect correlation between the data on each x- and y-axis (slope = 1). In **(A)**, global correlations between the changes induced by each antibiotic treatment are shown (*R*^2^ and *p*-values).

Expression of the *vanRSJKHAX* genes (SCO3589-SCO3596) comprising the VanB-type glycopeptide resistance cluster was significantly up-regulated in response to all three glycopeptide antibiotics, but on average was induced > 32 fold more strongly in response to vancomycin (Figure 3A and Supplementary Figure 1A). Chlorobiphenyl-vancomycin and dalbavancin stimulated similar levels of expression of the resistance system. At the global level, antibiotic-induced changes in transcript abundance relative to the control correlate highly between vancomycin and chlorobiphenyl-vancomycin (*R*^2^ = 0.739) and vancomycin and dalbavancin (*R*^2^ = 0.551) but the data for the *vanRSJKHAX* genes is notably deviant (Figure 3A). Vancomycin and chlorobiphenyl-vancomycin, however, produce similar responses in gene transcription controlled by the extracytoplasmic function sigma factor SigE that is a major system for responding to cell wall stress in *S. coelicolor* (Tran et al., 2019) (Figure 3B and Supplementary Figure 1B). The response of the *sigE* regulon to dalbavancin was also highly similar, but slightly less well conserved. In contrast, induction of a transcriptional response associated with zinc starvation stress through physicochemical interaction of vancomycin with Zn^2+^ (Zarkan et al., 2016, 2017) is largely absent in dalbavancin treated cultures and is attenuated in the response to chlorobiphenyl-vancomycin (Figure 3C).

### Transcripts Responding Significantly to Glycopeptide Antibiotic Treatment Tend Not to Be Leaderless

Previous mapping of transcriptional start sites in the *S. coelicolor* genome identified a notable proportion of gene transcripts, about 20%, that do not possess a 5′-UTR region but start at or adjacent to their translational start sites (Jeong et al., 2016). Such leaderless transcription of genes can impact both the transcriptional and post-transcriptional regulation of gene product formation. Genes whose transcription was significantly altered in response to glycopeptide antibiotic treatment and for which transcription start site data is available were analysed to determine any preference for leadered or leaderless transcription (Table 1). Interestingly, leaderless transcripts are significantly under-represented in the group of genes significantly up-regulated in response to all three glycopeptides and also in the groups down-regulated in response to two or more of the antibiotics used. In contrast, leaderless transcripts are significantly over-represented in the group of genes whose transcription was not changed significantly following exposure to any of the antibiotic treatments.

**TABLE 1:** Leaderless transcripts are significantly under-represented in genes responding to glycopeptide antibiotic stress.

Group	Leaderless*	With 5′-UTR*	Proportion Leaderless/total	Fisher’s test versus the universe group (*p*-value)	
				Greater	Lesser
Down in all 3	12	102	0.11	9.97e-01	**7.70e-03**
Down only in 2	12	104	0.10	9.97e-01	**6.16e-03**
Down only in 1	69	277	0.20	4.62e-01	5.94e-01
No change in all 3	426	1512	0.22	**1.97e-02**	9.83e-01
Up only in 1	102	376	0.21	2.00e-01	8.32e-01
Up only in 2	29	165	0.15	9.58e-01	6.33e-02
Up in all 3	58	369	0.14	9.91e-01	**1.24e-03**
Total	708	2905	0.20	-	-

*From transcription start site data in Jeong et al. (2016) supplementary file 2. The group membership data have been filtered to remove transcripts for which no primary promoter was mapped. The data factors in the operon membership of genes.

### Genes Down-Regulated by Exposure to Glycopeptide Antibiotics Are Significantly Enriched for Those Dependent on Sigma Factor HrdB for Their Transcription

The initiation of gene transcription in bacteria requires both RNA polymerase and a specific sigma factor that can recognise the promoter sequence and mediate initiation. The principal sigma factor in *S. coelicolor* is sigma factor HrdB and is required for cell viability. The regulon of genes controlled by HrdB includes those required for primary metabolism and vegetative cell growth (Smidova et al., 2019). Genes dependent on HrdB for their transcription are significantly over-represented in the groups of genes down-regulated in response to at least one of the glycopeptide antibiotics (Table 2), consistent with a decrease in active growth following the treatments. This corresponds with a significant > 2-fold decrease in transcription of sigma factor HrdB (SCO5820) itself in response to all the glycopeptides studied (Supplementary Data 1). Interestingly, HrdB-dependent transcripts are also significantly under-represented in the group of genes whose expression is not affected by any antibiotic treatment (Table 2).

**TABLE 2:** Transcripts down-regulated in response to glycopeptide antibiotic stress are significantly enriched for control by HrdB-dependent promoters.

Group	HrdB-dependent*	HrdB-independent*	Proportion HrdB/Total	Fisher’s test versus the universe group (*p*-value)	
				Greater	Lesser
Down in all 3	63	101	0.38	**1.47e-03**	9.99e-01
Down only in 2	80	136	0.37	**1.57e-03**	9.99e-01
Down only in 1	267	383	0.41	**4.29e-13**	1.00e + 00
No change in all 3	1155	3505	0.25	9.99e-01	**7.81e-04**
Up only in 1	262	678	0.28	3.90e-01	6.40e-01
Up only in 2	104	262	0.28	3.53e-01	6.90e-01
Up in all 3	166	495	0.25	9.05e-01	1.12e-01
Total	2097	5560	0.27	-	-

*From ChIP-seq data in Smidova et al. (2019) supplementary file 2. “HrdB-independence” was assigned to gene transcripts for which there was no reported evidence of binding of HrdB in 5′UTR regions. The data factors in the operon membership of genes.

### Different Glycopeptide Antibiotics Induce Distinct Changes in Gene Transcription Required for Amino Acid Biosynthesis

**FIGURE 4:**
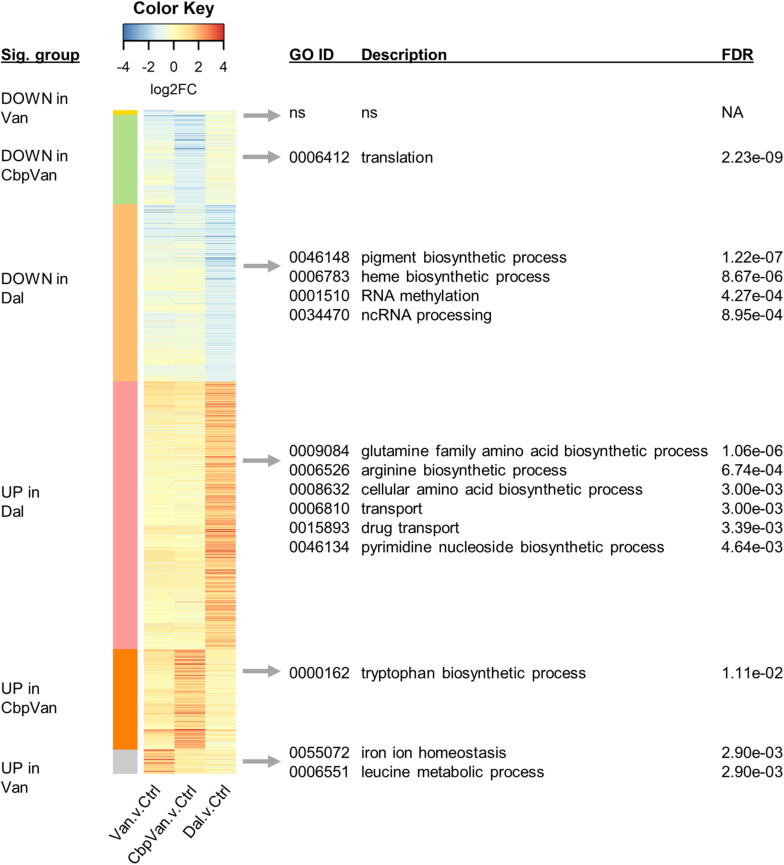
Heatmap summarising the transcripts uniquely changing significantly in response to only one of the glycopeptide antibiotics used, relative to the control treatment. Significant biological process categories from gene ontology (GO) functional enrichment analysis of each group of genes are shown to the right (“ns” denotes cases where no significant categories were identified). Complete GO enrichment analysis results are provided in Supplementary Data 2.

**FIGURE 5:**
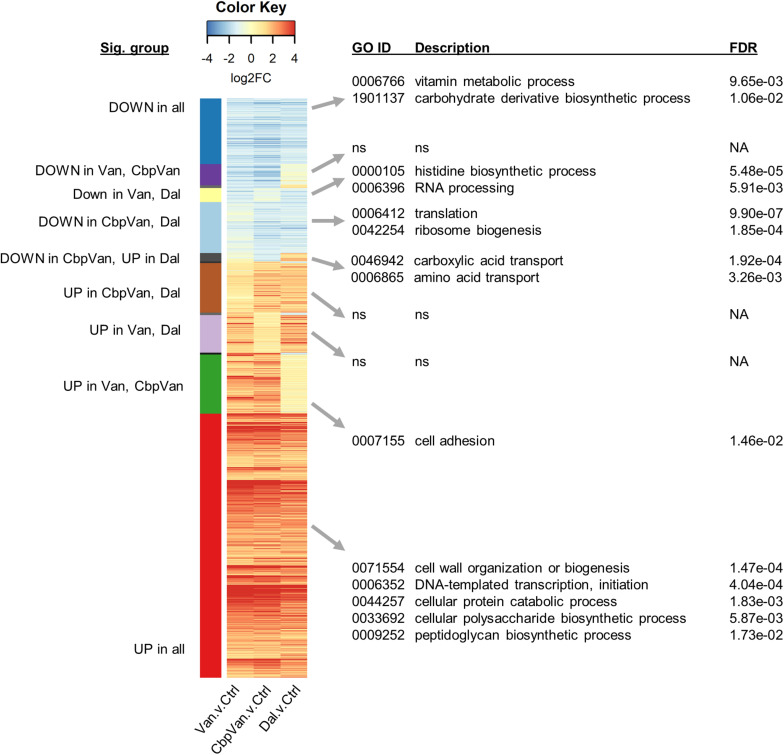
Heatmap summarising the transcripts changing significantly in response to two or more of the glycopeptide antibiotics used relative to the control treatment. Significant biological process categories from gene ontology (GO) functional enrichment analysis of each group of genes are shown to the right (“ns” denotes cases where no significant categories were identified). Only groups containing more than 10 transcripts are labelled. Complete GO enrichment analysis results are provided in Supplementary Data 2.

The significant changes in gene transcription occurring uniquely in response to only one of the three glycopeptide antibiotics studied are summarised in Figure 4, while significant responses to two or more antibiotics are presented in Figure 5. Functional enrichment analysis of the different groups of genes present in this data, based on the *S. coelicolor* gene ontology biological process (GO BP) annotation, highlights the cellular processes that are affected by the changes in gene expression observed (Figures 4, 5 and Supplementary Data 2). Strikingly, amino acid biosynthesis emerges as a process that is affected differently by each glycopeptide antibiotic. Genes uniquely up-regulated in response to dalbavancin are significantly enriched for amino acid biosynthesis (GO: 0008632 3.00e-03), predominantly those required for glutamate and arginine biosynthesis (GO:0009084 1.06e-06 and GO:0006526 6.74e-04, respectively) (Figure 4 and Supplementary Figure 2). In contrast, up-regulation of tryptophan biosynthesis is uniquely associated with chlorobiphenyl-vancomycin (GO:0000162 1.11e-02), and up-regulation of leucine metabolism is similarly associated with vancomycin treatment (GO:0006551 2.90e-03) (Figure 4 and Supplementary Figure 2). Genes required for histidine biosynthesis are significantly enriched in the group of genes down-regulated in response to both vancomycin and dalbavancin but not to chlorobiphenyl-vancomycin (GO:0000105 5.48e-05) (Figure 5 and Supplementary Figure 2).

### Dalbavancin Has Unique Effects on Cell Transport Processes, RNA Methylation and Haem and Pyrimidine Biosynthesis

Dalbavancin elicits the most extensive changes in gene transcription of all the glycopeptides studied, with 1109 genes uniquely up- or down-regulated (Figure 2C). The uniquely up-regulated genes are significantly enriched for transport functions (GO:0006810 3.00e-03) and include gene products required for amino acid import, and sugar, phosphate and oligopeptide transport (Figure 4 and Supplementary Figure 3). Genes required for pyrimidine nucleoside biosynthesis are also enriched in this category (GO:0046134 4.64e-03) (Figure 4 and Supplementary Figure 3). The uniquely down-regulated genes are significantly enriched for functions related to haem biosynthesis (GO:0006783 8.67e-06) and the methylation of RNA (GO:0001510 4.27e-04) (Figure 4 and Supplementary Figure 3).

### Gene Transcription Encoding Production of the Translational Apparatus Is More Extensively Repressed by Chlorobiphenyl-Vancomycin

The translational apparatus is considered a limiting factor for the growth of bacterial cells, such that growth rate is directly proportional to the number of ribosomes in a cell. Genes uniquely down-regulated in response to chlorobiphenyl-vancomycin are significantly associated with translation (GO:0006412 2.23e-09) suggesting a more pronounced effect on growth than vancomycin or dalbavancin (Figure 4 and Supplementary Figure 4A). A detrimental effect on ribosome biogenesis is largely absent in the response to vancomycin, but some down-regulation is evident following dalbavancin treatment. The group of genes significantly repressed in the presence of dalbavancin and chlorobiphenyl-vancomycin, but not vancomycin, is also significantly enriched for the translation GO BP category (GO:0006412 9.90e-07) (Figure 5 and Supplementary Figure 4B).

### Cellular Systems for the Reprogramming of Gene Transcription and for Bacterial Cell Wall Biogenesis Form Part of the Conserved Response to Glycopeptide Antibiotics

The response conserved between all three glycopeptide antibiotics studied included the significant induction of transcription of 661 genes and the concomitant repression of 164 genes (Figure 2C). Functional enrichment analysis of the up-regulated genes identifies a reorganisation of cellular metabolism associated with cell wall biogenesis, peptidoglycan synthesis, protein catabolism and polysaccharide biosynthesis (Figure 5). A GO category related to transcription initiation is also significantly over-represented in this up-regulated set of genes (GO:0006352 4.04e-04), and these correspond to 16 genes encoding alternative RNA polymerase sigma factors (Supplementary Figure 4C). Each alternative sigma factor recognises a different promoter sequence to that controlled by the primary sigma factor HrdB and thus each specifies initiation of transcription at distinct sets of genes different from the HrdB-dependent gene transcription that is predominantly required for primary metabolism and vegetative growth.

### Dynamic Transcription of Short RNAs Is a Feature of Glycopeptide Antibiotic Stress

**FIGURE 6:**
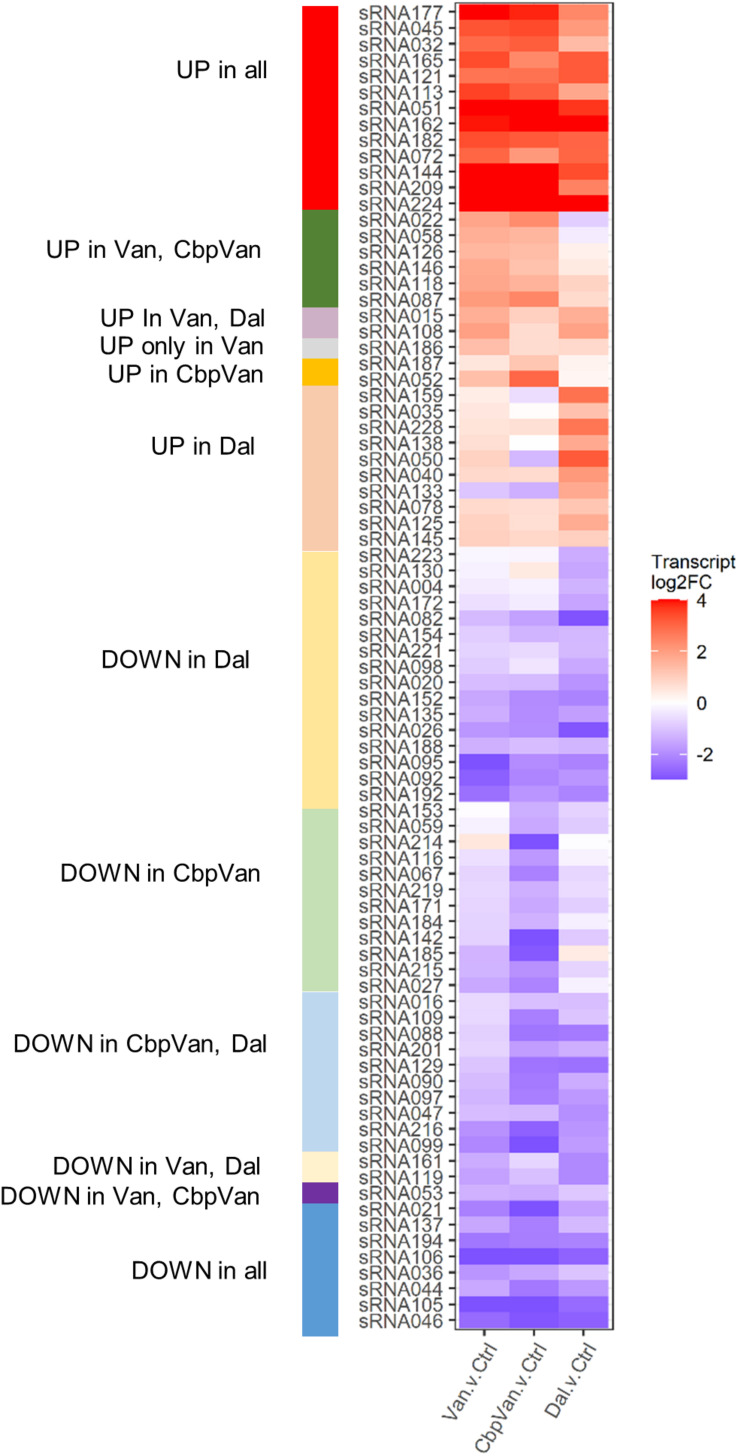
sRNAs responding significantly to glycopeptide antibiotic stress.

Short non-coding RNA transcripts (sRNAs) form part of the molecular machinery that regulates bacterial gene expression. Jeong et al. (Jeong et al., 2016) have previously identified 230 sRNAs in *S. coelicolor* that are putatively involved in transcriptional and post-transcriptional regulation of gene product formation, although their roles are not currently well understood. In this work, glycopeptide antibiotic stress caused significant changes in the transcription of 79 sRNAs (Figure 6). This included 21 that were reproducibly induced or repressed by all three glycopeptides studied, but also examples of sRNAs changing uniquely in response to only one.

## Discussion

Vancomycin, chlorobiphenyl-vancomycin and dalbavancin induce distinct transcriptional responses in the VanB-type resistant streptomycete *S. coelicolor*, despite sharing the same primary mode of antibiotic action and a high degree of chemical similarity. Vancomycin molecules bind Zn^2+^ ions via interaction with nitrogen and oxygen atoms in the N-terminal methylleucine moiety, a phenomenon initially observed due to the identification of a strong zinc-starvation response by exposure to vancomycin in *S. coelicolor* (Zarkan et al., 2016, 2017). The absence of a zinc-starvation response following dalbavancin treatment (Figure 3C) correlates with the absence of this methylleucine group in the structure of dalbavancin. Although the zinc-binding methylleucine feature is conserved in chlorobiphenyl-vancomycin, treatment with this glycopeptide produces a much weaker zinc-starvation response compared to vancomycin, presumably due to the presence of the bulky hydrophobic chlorobiphenyl group. Full interaction of vancomycin with Zn^2+^ proceeds via polymerisation of vancomycin dimers and the chlorobiphenyl group may compromise this dimerisation process. Interestingly, vancomycin also uniquely causes a significant up-regulation of a set of four transcription units encoding putative functions in iron ion homeostasis, suggesting a broader influence of the vancomycin structural features on metal ion homeostasis (Figure 4 and Supplementary Figure 4D).

The chlorobiphenyl group present in the glycopeptide antibiotic oritavancin is reported to perturb cell membrane integrity in Gram-positive bacteria through increased interaction with the lipid bilayer, providing an additional mode of antibiotic activity (Zhanel et al., 2012). While there was no direct evidence for membrane perturbation discernible in the transcriptome data characterising the response of *S. coelicolor* to chlorobiphenyl-vancomycin, a broader repressive effect on gene transcription required for production of the translational apparatus compared to both vancomycin and dalbavancin was detected (Figure 4 and Supplementary Figure 4). In addition, chlorobiphenyl-vancomycin instigated a unique up-regulation in genes annotated to tryptophan biosynthesis (Figure 4 and Supplementary Figure 2B). This corresponds to the *trp2* locus that is within the *cda* cluster of biosynthetic genes specifying production of the calcium-dependent antibiotic CDA and supplies tryptophan precursors for synthesis of CDA (Hojati et al., 2002). The *trp1* locus associated with tryptophan auxotrophy (SCO2036, SCO2037, SCO2039, SCO2043) was not up-regulated, but *trpA* (SCO2036) was uniquely significantly down-regulated in response to chlorobiphenyl-vancomycin (Supplementary Data 1). The switching on of CDA biosynthesis in *S. coelicolor* has been proposed to form part of a programmed cell death response (Novotna et al., 2003; Virolle, 2020), and it is interesting to note that significant up-regulation of the *cda* cluster genes occurs uniquely in response to chlorobiphenyl-vancomycin (Supplementary Figure 5A). A general down-regulation of translation coupled with activation of programmed cell death would be consistent with a more potent activity for chlorobiphenyl-vancomycin, perhaps related to membrane depolarisation.

Dalbavancin is the least structurally similar of the three antibiotics studied (Figure 1) and *S. coelicolor* cells exposed to dalbavancin were also the least similar at the level of their transcriptome profiles (Figure 2). Important structural features of dalbavancin include the long lipid side chain attached to the monosaccharide moiety and the dimethylaminopropyl amide side chain. The lipid side chain is associated with its activity against VanB-type resistant bacteria where its lipophilic interaction with the cell membrane is proposed to prevent sensing of the glycopeptide by the VanB-type VanS sensor kinase protein that resides in the membrane (Dong et al., 2002; Kwun and Hong, 2014). A marked induction of the VanS-dependent transcription of the *vanRSJKHAX* genes in the vancomycin resistance cluster in response to dalbavancin was still, however, observed in this study, but was ∼30-fold weaker than that produced by vancomycin itself. This reduced level of activation, also seen with chlorobiphenyl-vancomycin, is presumably insufficient to enable survival. Crystal structure analysis indicates that the positively charged dimethylaminopropyl amide group of dalbavancin remains free after interaction with its d-Ala–d-Ala target, permitting additional interactions to take place with negatively charged molecules such as phospholipid head groups of the bacterial membrane (Economou et al., 2012). Interestingly, transcripts uniquely up-regulated in response to dalbavancin are significantly enriched for transport processes, including the *pstABC* phosphate transport locus which is ∼2.5-fold induced (Supplementary Figure 3A). Such changes to activated transport mechanisms likely represent compensatory mechanisms acting against the reduced bioavailability of important nutrients or increased damage to the cell envelope. In addition to the general up-regulation of a broad range of transport systems, the dalbavancin response is also characterised by unique effects on amino acid biosynthesis, pyrimidine biosynthesis, RNA methylation and haem biosynthesis (Figure 4 and Supplementary Figures 2A, 3). While these effects are currently difficult to rationalise it is clear that the divergent structure of dalbavancin elicits a more divergent transcriptional response in *S. coelicolor*, relative to vancomycin or chlorobiphenyl-vancomycin.

A core signature response common to all three glycopeptides was evident, centred around activation of the SigE-regulated cell wall stress response, induction of genes encoding a raft of 16 alternative RNA polymerase sigma factors, an up-regulation of genes with functions related to cell wall biogenesis and reorganisation and an increase in transcripts encoding protein catabolic functions. Interestingly, leaderless transcripts were significantly under-represented amongst both the up and down regulated genes in the core response. Leaderless gene transcription is widespread in bacteria, especially in Actinobacteria where ∼20% of genes are leaderless (Zheng et al., 2011). Absence of a 5′-UTR leader sequence can impact the rate of transcript synthesis and degradation, through altered mRNA stability and reduced opportunities for interaction with regulatory sRNA molecules and also the rate of its translation, through a requirement for a different mechanism of translational initiation and again via the reduced opportunity for interactions with regulatory sRNA. A rigorous assessment of these features in *Mycobacerium smegmatis*, however, failed to identify a consistent global trend differentiating rates of transcription and translation by leadered or leaderless transcription (Nguyen et al., 2020). The observed preference for leadered transcripts in the induced response to glycopeptide antibiotic stress, and the enrichment of leaderless transcripts in those remaining unchanged following antibiotic challenge (Table 1), suggests that in *S. coelicolor* the presence of a 5′-UTR may give more reliable dynamic control over gene product formation. Interestingly, this is also observed in the response of *S. coelicolor* to heat shock stress (Bucca et al., 2018). The conserved elements of the response to all glycopeptides are perhaps surprising given the different outcomes of the different treatments: cells ultimately survive vancomycin exposure and recover active growth, while chlorobiphenyl-vancomycin and dalbavancin treatment leads to stasis and cell death. This is consistent with the selection of the 30 min time period to capture antibiotic-specific responses and avoid indirect effects: these fates have presumably yet to be arrived at in this study, and the cells are transitioning through a common response instigated to try to adapt and survive.

Transcripts significantly down-regulated in response to glycopeptide stress also tend to be dependent on the major vegetative sigma factor HrdB, while those not responding have a tendency to be HrdB-independent (Table 2). This is consistent with the significant down-regulation in transcription of *hrdB* (SCO5820) in response to all three glycopeptides. Concomitant up-regulation of expression of 16 alternative sigma factor genes, including *hrdD* (SCO3202) and eight extracytoplasmic function sigma factors, including *sigE* (SCO3356) and *bldN* (SCO3323) (Supplementary Figure 4C), can also be envisaged to decrease the proportion of RNA polymerase associating with HrdB and promote transcription initiation away from HrdB-controlled genes. The *S. coelicolor* genome encodes 65 sigma factors, an unusually large number for a bacterium, most of which are alternative sigma factors controlling transcription of regulons yet to be determined. The results here elucidate the biological role of some of these as stress induced alternative sigma factors. Interestingly, leaderless transcripts are significantly under-represented in HrdB-dependent transcripts (and conversely over-represented in those that are HrdB-independent) (Supplementary Table 2).

Assessing structure-activity relationships through genome-wide profiling depends on the quality of the genome annotation available for the reporter organism. For chemicals with activities against eukaryotic cells, the yeast *Saccharomyces cerevisiae* is used extensively for this purpose via chemogenomics techniques, relying on the existence of one of the most extensive functional annotations of any genome (Gasch et al., 2000; Giaever et al., 2004; Andrusiak et al., 2012; Lee et al., 2014). The annotation of bacterial genomes, with the possible exception of *E. coli*, has not yet reached the same level of maturity, and the genome of *S. coelicolor* is limited both by the presence of a large proportion of genes with no known function and the dependence on computationally inferring functions based on the annotations of homologs present in better studied organisms. Despite these limitations, the current study was able to usefully differentiate the activities of three closely related glycopeptide antibiotic structures.

## Conclusion

Transcriptome profiling has a track record in distinguishing bacterial responses to chemically very diverse antibiotics from different mode of action groups. Here we show that it can also usefully be employed to study structure-activity relationships within very closely related compounds possessing the same principal mode of action. The divergent responses observed for the three different glycopeptide antibiotics tested, centred around a core set of changes common to all, indicate that in addition to their shared mode of action, each can have its own additional influences on bacterial physiology that are related to the peripheral differences in their structures. While only a partial interpretation of these differences has been possible – limited by an incomplete understanding of the functional role of all the genes in the *S. coelicolor* genome – this work indicates the utility of the approach. As the functional annotation of bacterial genomes matures, finer grained interpretations of structure-activity relationships will become possible, providing important context for efforts aimed at designing modifications to improve the activities of useful antibiotics.

## Data Availability Statement

This data is available in the ArrayExpress database (http://www.ebi.ac.uk/arrayexpress) under accession number E-MTAB-9846.

## Author Contributions

H-JH and CS conceived and designed the study. H-JH performed the antibiotic challenge experiments. GB performed the transcriptome sequencing. AH undertook the data processing and analysis. All authors contributed to the writing of the manuscript and to interpretation of the data.

## Conflict of Interest

The authors declare that the research was conducted in the absence of any commercial or financial relationships that could be construed as a potential conflict of interest.

## Footnotes

1 http://www.ebi.ac.uk/arrayexpress

## References

[B1] Andrews S. (2010). *fastqc: A Quality Control Tool for High Throughput Sequence Data* (v.0.11.8). Available online at: http://www.bioinformatics.babraham.ac.uk/projects/fastqc/

[B2] Andrusiak K. Piotrowski J. S. Boone C. (2012). Chemical-genomic profiling: systematic analysis of the cellular targets of bioactive molecules. *Bioorg. Med. Chem.* 20 1952–1960. 10.1016/j.bmc.2011.12.023 22261022

[B3] Barna J. C. Williams D. H. (1984). The structure and mode of action of glycopeptide antibiotics of the vancomycin group. *Annu. Rev. Microbiol.* 38 339–357. 10.1146/annurev.mi.38.100184.002011 6388496

[B4] Blaskovich M. A. T. Hansford K. A. Butler M. S. Jia Z. Mark A. E. Cooper M. A. (2018). Developments in glycopeptide antibiotics. *ACS Infect. Dis.* 4 715–735. 29363950 10.1021/acsinfecdis.7b00258 PMC5952257

[B5] Boshoff H. I. Myers T. G. Copp B. R. McNeil M. R. Wilson M. A. Barry C. E. III (2004). The transcriptional responses of *Mycobacterium tuberculosis* to inhibitors of metabolism: novel insights into drug mechanisms of action. *J. Biol. Chem.* 279 40174–40184. 15247240 10.1074/jbc.M406796200

[B6] Bray N. L. Pimentel H. Melsted P. Pachter L. (2016). Near-optimal probabilistic RNA-seq quantification. *Nat. Biotechnol.* 34 525–527. 10.1038/nbt.3519 27043002

[B7] Bucca G. Pothi R. Hesketh A. Moller-Levet C. Hodgson D. A. Laing E. E. (2018). Translational control plays an important role in the adaptive heat-shock response of *Streptomyces coelicolor*. *Nucleic Acids Res.* 46 5692–5703. 10.1093/nar/gky335 29746664 PMC6009599

[B8] Bugg T. D. Dutka-Malen S. Arthur M. Courvalin P. Walsh C. T. (1991a). Identification of vancomycin resistance protein VanA as a D-alanine:D-alanine ligase of altered substrate specificity. *Biochemistry* 30 2017–2021. 10.1021/bi00222a002 1998664

[B9] Bugg T. D. Wright G. D. Dutka-Malen S. Arthur M. Courvalin P. Walsh C. T. (1991b). Molecular basis for vancomycin resistance in *Enterococcus faecium* BM4147: biosynthesis of a depsipeptide peptidoglycan precursor by vancomycin resistance proteins VanH and VanA. *Biochemistry* 30 10408–10415. 10.1021/bi00107a007 1931965

[B10] Butler M. S. Hansford K. A. Blaskovich M. A. Halai R. Cooper M. A. (2014). Glycopeptide antibiotics: back to the future. *J. Antibiot. (Tokyo)* 67 631–644. 10.1038/ja.2014.111 25118105

[B11] CDC (2019). *Antibiotic Resistance Threats in the United States.* Atlanta, GA: CDC.

[B12] Charaniya S. Mehra S. Lian W. Jayapal K. P. Karypis G. Hu W. S. (2007). Transcriptome dynamics-based operon prediction and verification in *Streptomyces coelicolor*. *Nucleic Acids Res.* 35 7222–7236. 10.1093/nar/gkm501 17959654 PMC2175336

[B13] Cui L. Ma X. Sato K. Okuma K. Tenover F. C. Mamizuka E. M. (2003). Cell wall thickening is a common feature of vancomycin resistance in *Staphylococcus aureus*. *J. Clin. Microbiol.* 41 5–14. 10.1128/jcm.41.1.5-14.2003 12517819 PMC149586

[B14] Dong S. D. Oberthur M. Losey H. C. Anderson J. W. Eggert U. S. Peczuh M. W. (2002). The structural basis for induction of VanB resistance. *J. Am. Chem. Soc.* 124 9064–9065. 10.1021/ja026342h 12149006

[B15] Economou N. J. Nahoum V. Weeks S. D. Grasty K. C. I Zentner J. Townsend T. M. (2012). A carrier protein strategy yields the structure of dalbavancin. *J. Am. Chem. Soc.* 134 4637–4645. 10.1021/ja208755j 22352468 PMC3304006

[B16] Ewels P. Magnusson M. Lundin S. Kaller M. (2016). MultiQC: summarize analysis results for multiple tools and samples in a single report. *Bioinformatics* 32 3047–3048. 10.1093/bioinformatics/btw354 27312411 PMC5039924

[B17] Gasch A. P. Spellman P. T. Kao C. M. Carmel-Harel O. Eisen M. B. Storz G. (2000). Genomic expression programs in the response of yeast cells to environmental changes. *Mol. Biol. Cell* 11 4241–4257. 10.1091/mbc.11.12.4241 11102521 PMC15070

[B18] Giaever G. Flaherty P. Kumm J. Proctor M. Nislow C. Jaramillo D. F. (2004). Chemogenomic profiling: identifying the functional interactions of small molecules in yeast. *Proc. Natl. Acad. Sci. U.S.A.* 101 793–798. 10.1073/pnas.0307490100 14718668 PMC321760

[B19] Hanaki H. Kuwahara-Arai K. Boyle-Vavra S. Daum R. S. Labischinski H. Hiramatsu K. (1998a). Activated cell-wall synthesis is associated with vancomycin resistance in methicillin-resistant *Staphylococcus aureus* clinical strains Mu3 and Mu50. *J. Antimicrob. Chemother.* 42 199–209. 10.1093/jac/42.2.199 9738837

[B20] Hanaki H. Labischinski H. Inaba Y. Kondo N. Murakami H. Hiramatsu K. (1998b). Increase in glutamine-non-amidated muropeptides in the peptidoglycan of vancomycin-resistant *Staphylococcus aureus* strain Mu50. *J. Antimicrob. Chemother.* 42 315–320. 10.1093/jac/42.3.315 9786471

[B21] Hastings J. Owen G. Dekker A. Ennis M. Kale N. Muthukrishnan V. (2016). ChEBI in 2016: improved services and an expanding collection of metabolites. *Nucleic Acids Res.* 44 D1214–D1219. 26467479 10.1093/nar/gkv1031 PMC4702775

[B22] Hojati Z. Milne C. Harvey B. Gordon L. Borg M. Flett F. (2002). Structure, biosynthetic origin, and engineered biosynthesis of calcium-dependent antibiotics from *Streptomyces coelicolor*. *Chem. Biol.* 9 1175–1187. 10.1016/s1074-5521(02)00252-1 12445768

[B23] Hong H. J. Hutchings M. I. Hill L. M. Buttner M. J. (2005). The role of the novel Fem protein VanK in vancomycin resistance in *Streptomyces coelicolor*. *J. Biol. Chem.* 280 13055–13061. 10.1074/jbc.m413801200 15632111

[B24] Hong H. J. Hutchings M. I. Neu J. M. Wright G. D. Paget M. S. Buttner M. J. (2004). Characterization of an inducible vancomycin resistance system in *Streptomyces coelicolor* reveals a novel gene (vanK) required for drug resistance. *Mol. Microbiol.* 52 1107–1121. 10.1111/j.1365-2958.2004.04032.x 15130128

[B25] Howden B. P. Davies J. K. Johnson P. D. Stinear T. P. Grayson M. L. (2010). Reduced vancomycin susceptibility in *Staphylococcus aureus*, including vancomycin-intermediate and heterogeneous vancomycin-intermediate strains: resistance mechanisms, laboratory detection, and clinical implications. *Clin. Microbiol. Rev.* 23 99–139. 10.1128/cmr.00042-09 20065327 PMC2806658

[B26] Hutchings M. I. Hong H. J. Buttner M. J. (2006). The vancomycin resistance VanRS two-component signal transduction system of *Streptomyces coelicolor*. *Mol. Microbiol.* 59 923–935. 10.1111/j.1365-2958.2005.04953.x 16420361

[B27] Hutter B. Schaab C. Albrecht S. Borgmann M. Brunner N. A. Freiberg C. (2004). Prediction of mechanisms of action of antibacterial compounds by gene expression profiling. *Antimicrob. Agents Chemother.* 48 2838–2844. 10.1128/aac.48.8.2838-2844.2004 15273089 PMC478524

[B28] Jeong Y. Kim J. N. Kim M. W. Bucca G. Cho S. Yoon Y. J. (2016). The dynamic transcriptional and translational landscape of the model antibiotic producer *Streptomyces coelicolor* A3(2). *Nat. Commun.* 7:11605. 27251447 10.1038/ncomms11605 PMC4895711

[B29] Jones M. B. Nierman W. C. Shan Y. Frank B. C. Spoering A. Ling L. (2017). Reducing the bottleneck in discovery of novel antibiotics. *Microb. Ecol.* 73 658–667. 10.1007/s00248-016-0889-3 27896376

[B30] Kwun M. J. Hong H. J. (2014). The activity of glycopeptide antibiotics against resistant bacteria correlates with their ability to induce the resistance system. *Antimicrob. Agents Chemother.* 58 6306–6310. 10.1128/aac.03668-14 25092694 PMC4187914

[B31] Lee A. Y. St Onge R. P. Proctor M. J. I Wallace M. Nile A. H. Spagnuolo P. A. (2014). Mapping the cellular response to small molecules using chemogenomic fitness signatures. *Science* 344 208–211. 24723613 10.1126/science.1250217 PMC4254748

[B32] Marcone G. L. Binda E. Berini F. Marinelli F. (2018). Old and new glycopeptide antibiotics: from product to gene and back in the post-genomic era. *Biotechnol. Adv.* 36 534–554. 10.1016/j.biotechadv.2018.02.009 29454983

[B33] McCarthy D. J. Smyth G. K. (2009). Testing significance relative to a fold-change threshold is a TREAT. *Bioinformatics* 25 765–771. 10.1093/bioinformatics/btp053 19176553 PMC2654802

[B34] McCormick M. H. McGuire J. M. Pittenger G. E. Pittenger R. C. Stark W. M. (1955). Vancomycin, a new antibiotic. I. Chemical and biologic properties. *Antibiot. Annu.* 3 606–611. 13355336

[B35] Mishra N. M. I Stolarzewicz D. Cannaerts J. Schuermans R. Lavigne Y. Looz B. (2018). Iterative chemical engineering of vancomycin leads to novel vancomycin analogs with a high in vitro therapeutic index. *Front. Microbiol.* 9:1175. 10.3389/fmicb.2018.01175 29930540 PMC6001238

[B36] Morgan M. Shepherd L. (2020). *AnnotationHub: Client to Access AnnotationHub Resources. R package Version 2.20.0.*

[B37] Nguyen T. G. Vargas-Blanco D. A. Roberts L. A. Shell S. S. (2020). The impact of leadered and leaderless gene structures on translation efficiency, transcript stability, and predicted transcription rates in *Mycobacterium smegmatis*. *J. Bacteriol.* 202:e00746-19. 32094162 10.1128/JB.00746-19 PMC7148126

[B38] Novotna J. Vohradsky J. Berndt P. Gramajo H. Langen H. Li X. M. (2003). Proteomic studies of diauxic lag in the differentiating prokaryote *Streptomyces coelicolor* reveal a regulatory network of stress-induced proteins and central metabolic enzymes. *Mol. Microbiol.* 48 1289–1303. 10.1046/j.1365-2958.2003.03529.x 12787356

[B39] Okano A. Isley N. A. Boger D. L. (2017). Total syntheses of vancomycin-related glycopeptide antibiotics and key analogues. *Chem. Rev.* 117 11952–11993. 10.1021/acs.chemrev.6b00820 28437097 PMC5617763

[B40] Okano A. Nakayama A. Schammel A. W. Boger D. L. (2014). Total synthesis of [Psi[C(horizontal lineNH)NH]Tpg(4)]vancomycin and its (4-chlorobiphenyl)methyl derivative: impact of peripheral modifications on vancomycin analogues redesigned for dual D-Ala-D-Ala and D-Ala-D-Lac binding. *J. Am. Chem. Soc.* 136 13522–13525. 10.1021/ja507009a 25211770 PMC4183650

[B41] O’Rourke A. Beyhan S. Choi Y. Morales P. Chan A. P. Espinoza J. L. (2020). Mechanism-of-action classification of antibiotics by global transcriptome profiling. *Antimicrob. Agents Chemother.* 64:e01207-19. 31907190 10.1128/AAC.01207-19 PMC7038283

[B42] Pootoolal J. Neu J. Wright G. D. (2002). Glycopeptide antibiotic resistance. *Annu. Rev. Pharmacol. Toxicol.* 42 381–408. 11807177 10.1146/annurev.pharmtox.42.091601.142813

[B43] R Core Team (2020). *R: A Language and Environment For Statistical Computing.* Vienna: R Foundation for Statistical Computing.

[B44] Ritchie M. E. Phipson B. Wu D. Hu Y. Law C. W. Shi W. (2015). limma powers differential expression analyses for RNA-sequencing and microarray studies. *Nucleic Acids Res.* 43:e47. 10.1093/nar/gkv007 25605792 PMC4402510

[B45] Santos-Beneit F. (2018). Genome sequencing analysis of *Streptomyces coelicolor* mutants that overcome the phosphate-depending vancomycin lethal effect. *BMC Genomics* 19:457. 10.1186/s12864-018-4838-z 29898657 PMC6001138

[B46] Santos-Beneit F. Martin J. F. (2013). Vancomycin resistance in *Streptomyces coelicolor* is phosphate-dependent but is not mediated by the PhoP regulator. *J. Glob. Antimicrob. Resist.* 1 109–113. 10.1016/j.jgar.2013.03.003 27873577

[B47] Smidova K. Zikova A. Pospisil J. Schwarz M. Bobek J. Vohradsky J. (2019). DNA mapping and kinetic modeling of the HrdB regulon in *Streptomyces coelicolor*. *Nucleic Acids Res.* 47 621–633. 10.1093/nar/gky1018 30371884 PMC6344877

[B48] Tailhades J. Zhao Y. Ho Y. T. C. Greule A. I Schoppet A. M. Kulkarni K. (2020). A chemoenzymatic approach to the synthesis of glycopeptide antibiotic analogues. *Angew. Chem. Int. Ed. Engl.* 59 10899–10903. 10.1002/anie.202003726 32297389

[B49] Tran N. T. Huang X. Hong H. J. Bush M. J. Chandra G. Pinto D. (2019). Defining the regulon of genes controlled by sigma(E), a key regulator of the cell envelope stress response in *Streptomyces coelicolor*. *Mol. Microbiol.* 112 461–481. 10.1111/mmi.14250 30907454 PMC6767563

[B50] Virolle M. J. (2020). A challenging view: antibiotics play a role in the regulstion of the energetic metabolism of the producing bacteria. *Antibiotics (Basel)* 9:83. 10.3390/antibiotics9020083 32069930 PMC7168255

[B51] Walsh C. T. Fisher S. L. I Park S. Prahalad M. Wu Z. (1996). Bacterial resistance to vancomycin: five genes and one missing hydrogen bond tell the story. *Chem. Biol.* 3 21–28. 10.1016/s1074-5521(96)90079-4 8807824

[B52] Xu M. Wang W. Waglechner N. Culp E. J. Guitor A. K. Wright G. D. (2020). GPAHex-A synthetic biology platform for Type IV-V glycopeptide antibiotic production and discovery. *Nat. Commun.* 11:5232. 33067466 10.1038/s41467-020-19138-5 PMC7567792

[B53] Yarlagadda V. Sarkar P. Manjunath G. B. Haldar J. (2015). Lipophilic vancomycin aglycon dimer with high activity against vancomycin-resistant bacteria. *Bioorg. Med. Chem. Lett.* 25 5477–5480. 10.1016/j.bmcl.2015.10.083 26525861

[B54] Yu G. Wang L. G. Han Y. He Q. Y. (2012). clusterProfiler: an R package for comparing biological themes among gene clusters. *OMICS* 16 284–287. 10.1089/omi.2011.0118 22455463 PMC3339379

[B55] Zarkan A. Macklyne H. R. Chirgadze D. Y. Bond A. D. Hesketh A. R. Hong H. J. (2017). Zn(II) mediates vancomycin polymerization and potentiates its antibiotic activity against resistant bacteria. *Sci. Rep.* 7:4893. 28687742 10.1038/s41598-017-04868-2 PMC5501778

[B56] Zarkan A. Macklyne H. R. Truman A. W. Hesketh A. R. Hong H. J. (2016). The frontline antibiotic vancomycin induces a zinc starvation response in bacteria by binding to Zn(II). *Sci. Rep.* 6:19602. 26797186 10.1038/srep19602 PMC4726154

[B57] Zhanel G. G. Schweizer F. Karlowsky J. A. (2012). Oritavancin: mechanism of action. *Clin. Infect. Dis.* 54 (Suppl. 3) S214–S219. 22431851 10.1093/cid/cir920

[B58] Zheng X. Hu G. Q. She Z. S. Zhu H. (2011). Leaderless genes in bacteria: clue to the evolution of translation initiation mechanisms in prokaryotes. *BMC Genomics* 12:361. 10.1186/1471-2164-12-361 21749696 PMC3160421

