# Supplementary material for: Chemotranscriptomic Profiling Defines Drug-Specific Signatures of the Glycopeptide Antibiotics Dalbavancin, Vancomycin and Chlorobiphenyl-Vancomycin in a VanB-Type-Resistant Streptomycete
Source: Front Microbiol. 2021 Feb 25;12:641756. doi: 10.3389/fmicb.2021.641756 (PMC7947799; doi:10.3389/fmicb.2021.641756)
Supplement: Supplementary file 3 [file Table_3.DOCX]

Supplementary Table 1. Classification of gene transcripts according to their combined response to each glycopeptide antibiotic.

| Group | Response to vancomycin* | Response to chlorobiphenyl vancomycin* | Response to dalbavancin* | Number of transcripts in group |
| --- | --- | --- | --- | --- |
| Down in all | -1 | -1 | -1 | 164 |
| Down Van, CbpVan | -1 | -1 | 0 | 53 |
| Down Van & CbpVan, Up Dal | -1 | -1 | 1 | 7 |
| Down Van, Dal | -1 | 0 | -1 | 35 |
| Down Van | -1 | 0 | 0 | 11 |
| Down CbpVan, Dal | 0 | -1 | -1 | 128 |
| Down CbpVan | 0 | -1 | 0 | 214 |
| Down CbpVan, Up Dal | 0 | -1 | 1 | 21 |
| Down Dal | 0 | 0 | -1 | 425 |
| No change any | 0 | 0 | 0 | 4660 |
| Up Dal | 0 | 0 | 1 | 641 |
| Up CbpVan, Down Dal | 0 | 1 | -1 | 4 |
| Up CbpVan | 0 | 1 | 0 | 241 |
| Up CbpVan, Dal | 0 | 1 | 1 | 124 |
| Up Van, Down Dal | 1 | 0 | -1 | 6 |
| Up Van | 1 | 0 | 0 | 58 |
| Up Van, Dal | 1 | 0 | 1 | 94 |
| Up Van & CbpVan, Down Dal | 1 | 1 | -1 | 5 |
| Up Van, CbpVan | 1 | 1 | 0 | 148 |
| Up in all | 1 | 1 | 1 | 661 |

* 1, significantly up-regulated; -1, significantly down-regulated; 0, no significant change

Supplementary Table 2. Leaderless transcripts are significantly under-represented in transcripts dependent on sigma HrdB for transcript initiation, and over-represented in HrdB-independent transcripts.

| Group# | Leaderless* | With 5’-UTR* | Proportion  Leaderless/Total | Fisher’s test versus the universe group  (p-value) | |
| --- | --- | --- | --- | --- | --- |
|  |  |  |  | greater | lesser |
| HrdB-dependent | 228 | 1283 | 0.15 | 1.00e+00 | **9.65e-05** |
| HrdB-independent | 482 | 1652 | 0.23 | **2.77e-03** | 9.98e-01 |
| Total | 710 | 2935 | 0.19 | - | - |

#From ChIP-seq data in Smidova et al. (2019) Supplementary file 2. “HrdB-independence” was assigned to gene transcripts for which there was no reported evidence of binding of HrdB in 5’-UTR regions. The data factors in operon membership of genes.

*From transcription start site data in Jeong et al. (2016) Supplementary file 2. The group membership data has been filtered to remove transcripts for which no primary promoter was mapped. The data factors in operon membership of genes.
